# Application of a CNN to the Boda Claystone Formation for high-level radioactive waste disposal

**DOI:** 10.1038/s41598-023-31564-1

**Published:** 2023-04-04

**Authors:** Virág Lovász, Amadé Halász, Péter Molnár, Róbert Karsa, Ákos Halmai

**Affiliations:** 1 Doctoral School of Earth Sciences, Faculty of Sciences, University of Pécs, Ifjúság útja 6., Pécs, 7624 Hungary; 2 Institute of Geography and Earth Sciences, Faculty of Sciences, University of Pécs, Ifjúság útja 6., Pécs, 7624 Hungary; 3 Public Limited Company for Radioactive Waste Management (PURAM), Esztergár Lajos u. 19., Pécs, 7633 Hungary; 4 Baranya County Disaster Management Directorate, Engel János József u. 1., Pécs, 7630 Hungary

**Keywords:** Environmental impact, Structural geology, Computational science

## Abstract

Nations relying on nuclear power generation face great responsibilities when designing their firmly secured final repositories. In Hungary, the potential host rock [the Boda Claystone Formation (BCF)] of the deep geological repository is under extensive examination. To promote a deeper comprehension of potential radioactive isotope transport and ultimately synthesis for site evaluation purposes, we have efficiently tailored geospatial image processing using a convolutional neural network (CNN). We customized the CNN according to the intricate nature of the fracture geometries in the BCF, enabling the recognition process to be particularly sensitive to details and to interpret them in the correct tectonic context. Furthermore, we set the highest processing scale standards to measure the performance of our model, and the testing circumstances intentionally involved various technological and geological hindrances. Our presented model reached ~ 0.85 precision, ~ 0.89 recall, an ~ 0.87 F1 score, and a ~ 2° mean error regarding dip value extraction. With the combination of a CNN and geospatial methodology, we present the description, performance, and limits of a fully automated workflow for extracting BCF fractures and their dipping data from scanned cores.

## Introduction

### Relevance of the research topic

Operating a nuclear power plant raises concerns about its dangerous byproduct: high-level, long-lived radioactive waste. Any kind of radioactive waste must be treated with great caution to safeguard both nature and human habitats^1,2^. The national responsibilities and challenges faced by the involved countries are particularly significant regarding the final repositories^2,3^.

As solutions for storing high-level radioactive material in the long term, deep geological repositories (DGRs) are highly supported by expertise^2,4–6^. To devise a DGR, the primary requirement is an available, suitable rock that can enable permanently stable isolation when supplemented with additional, engineered barriers^2^. The International Atomic Energy Agency (IAE) recommends executing the designation process in multiple stages to ensure its safe implementation in all respects^2^.

In Hungary, geological formations were specifically inspected as targets for the implementation of a DGR. The outcome pointed to the Boda Claystone Formation (at the base of the Mecsek Mountains) as a DGR option within the country^7,8^. Several boreholes have been deepened in the relevant expanse by the PURAM to collect BCF core samples and to put a diverse range of geological investigations into operation^7,9^. Following the IAE recommendation^2^, the implementation of an underground facility is among the long-term intents of the PURAM project^7^ to promote straight, on-the-spot investigation of the BCF^2,7^. To date, the Public Limited Company for Radioactive Waste Management (PURAM) has been running its large-scale planning program for decades, focusing on the yet-to-be-finalized function of the BCF^7–11^.

According to current knowledge, the spatial extent of the BCF is close to 150 km^2^^7,10,11^. This formation with a Permian, playa lake origin has a vertical extent of more than 800 m in thickness^7,10,11^. In general, the Boda Claystone is characterized by a grain size that is fining upward, consisting of reddish brown sandstone, siltstone, clayey siltstone, mudstone, and dolomite^10,12^.

The scientific literature extensively describes the low porosity and low permeability of this rock^11,13,14^. However, the presence of tectonic fractures somewhat modulates the latter^11^. The mineral and chemical composition of the BCF are excellent fluid flow inhibitors because the presence of swelling clay minerals results in the fractures possessing a natural self-sealing capability^14,15^. Nevertheless, fractures and inhomogeneities can still pose risks when transporting radioactive materials^14,16,17^. Fracture investigations promote the understanding of the hypothetical transport of radioactive isotopes, which is a pivotal issue in DGR safety^7,13,18,19^.

Furthermore, a comprehensive survey of all geological forces and influences is greatly needed to ensure safety in all regards^20^. Accordingly, several scientific articles regarding the investigation of BCF fractures and their fracture system have emerged, and this topic remains particularly important for the researchers involved^11,17,21^. Tectonic fractures in the BCF may occur in open or closed forms, which can be filled to varying degrees depending on additional processes^21,22^. In either case, they are meaningful sources of geological and geodynamical information not only on fluid flow but also on stability, structural development, tectonic prediction, and various relevant processes^14,16,18,23^. In these studies, dip, orientation, distribution, and fracture density have been among the extensively analyzed phenomena^19,21^.

Tóth et al. (2022a, 2022b) investigated the fracture network of the formation and carried out hydrogeological fracture modeling^13,19^. Fractures with or without any degree of filling were included in these investigations alike^19^. Furthermore, the dip, distribution, density, and formation of fractures are closely linked to paleotectonic stress fields, allowing us to reconstruct past movements^24,25^. Hrabovszki et al. (2017) investigated the BCF based on individual fracture geometries and dip angles, providing new information for reconstructing the structural evolution of the formation. Data on the densities, distributions, dip angles, and directions of individual fractures are essential for understanding these systems^14^. Accordingly, these studies supply great value for comprehensive site evaluations^7,13,18^. Although it is time- and labor-extensive, the registration of each individual fracture is a fundamental preliminary step for preparing these studies^18,22^. Among other geological phenomena, fractures, their dips and their depth values must be registered by the PURAM^22^. Substitutional solutions assisted with artificial intelligence can ease the fracture extraction process for the outlined investigations and documentation^18,26^.

**Figure 1:**
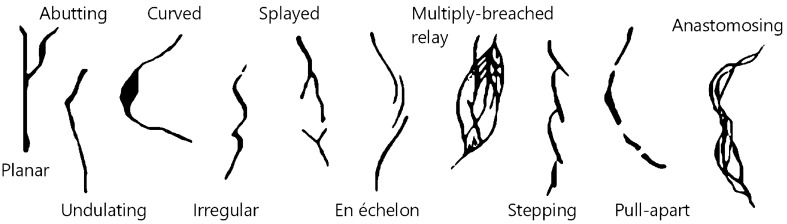
Typical fracture phenomena in the BCF. These are based on^29^ and^30^ and PURAM-owned scanned cores.

Detection is challenging due to the presence of various shapes, the continuity of the individual BCF fractures, and their occasionally diverse intersections^14,27–29^. Simplified drafts and nomenclature for the most elementary fractures occurring in the BCF are shown in Fig. 1.

Both the individual and relational characteristics are diverse; e.g., single fractures are present^19^, but these fractures can intersect one another as well. Moreover, individual fractures can exist in multiple parts^14,27–29^. Consequently, their corresponding vector-based forms in a geographical information system (GIS) database can include multipart polygons with intersections^31,32^.

**Figure 2:**
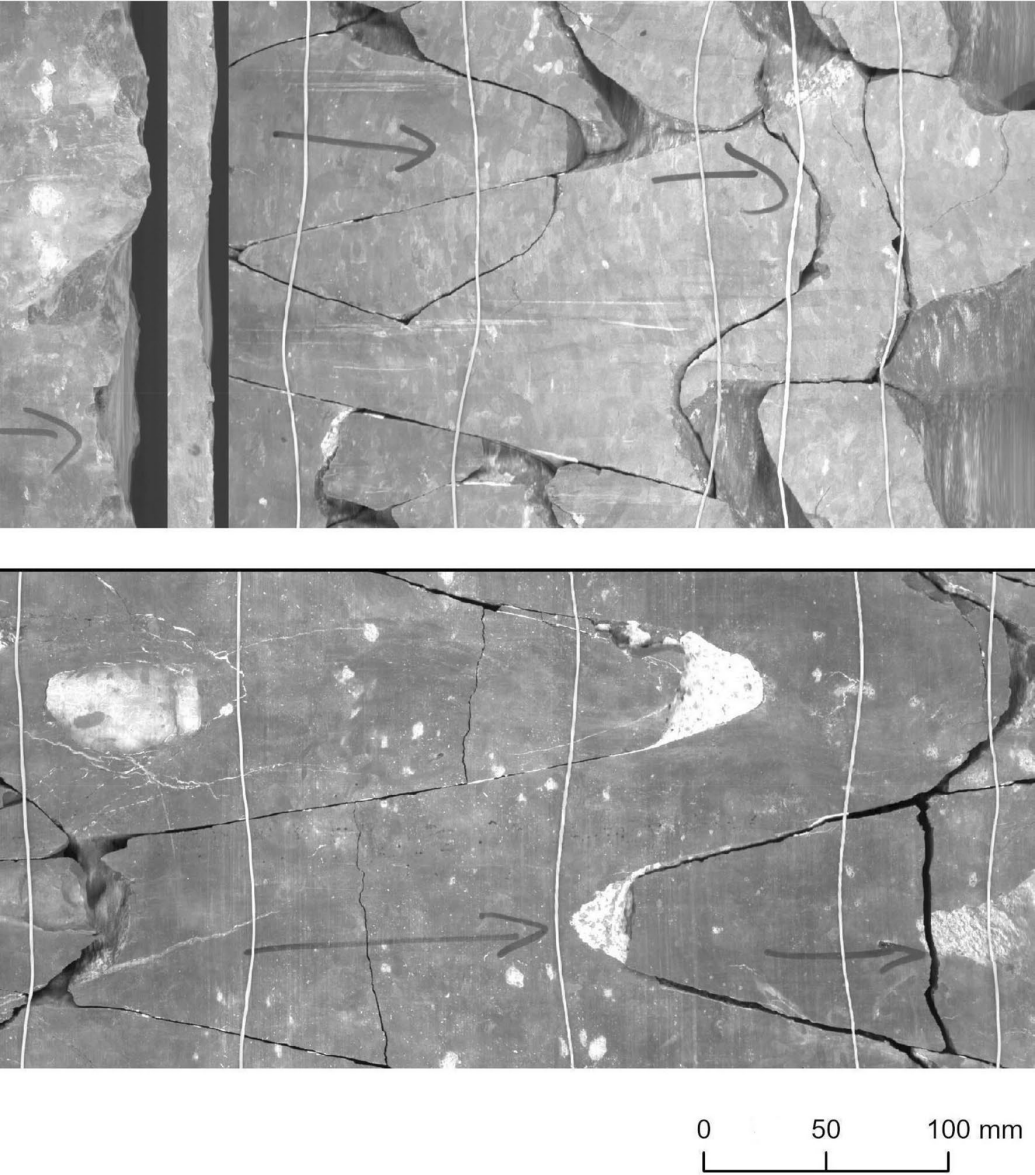
Undulating and planar-type fractures. Based on PURAM data.

BCF cores are scanned with the special method of the ImaGeo system^21,33^. Figure 2 shows the extended mantle of a scanned core, which explains why we see the individual fractures as sine curves. These sine-like lines represent natural fractures^21,33^. The white, almost parallel lines are rubber bands that hold the core together during the scanning process^33^.

The barely visible or even technological fractures make both recognition and correct differentiation difficult^14,22,34^.

Detection alone is not sufficient; this process should be done in a way that is authentic to the geologic interpretation. Each extracted fracture reflects the given tectonic process only if the model correctly recognizes the connected parts (e.g., in the case of a multipart fracture) or, where appropriate, separates the fractures from each other (in the case of intersection/tectonic subprocess) and if delineation is accurately performed on the sample^22,35^. These concerns are essential during manual documentation, but due to prioritization, certain details may also be omitted^22,35^. However, an automated model can be expected to eliminate this limitation and increase the level of detail in the produced documentation.

Deep learning can enrich geospatial strategies, exploiting the potential of semantic segmentation, instance segmentation, and object detection^36^. Since tectonic fractures can be subtle in terms of detection and their complexity and nuances are often not comparable to linear processes, artificial intelligence and neural networks are more suited for this task^18^. Convolutional neural networks (CNNs) have proven to be competent for handling diverse sets of geology-related image processing tasks, e.g., lithology classification, oil spill recognition^37,38^, or fault and horizon detection through seismic data^39^. The objective of our research was to test the feasibility of a CNN to recognize BCF fractures on scanned core samples to automate both their detection and dip calculation in a geospatial database.

### Objectives

Based on the necessities outlined in section “Relevance of the research topic”, there is a demand to utilize a CNN in a way that makes it perform efficiently on scanned BCF core samples, automating both the detection of fractures and the extraction of their dip values. We describe an applied methodology for this task, examining its possibilities and limits in detail. In this study, we aimed for the CNN to address predominantly unfilled fractures. To simplify the rest of the manuscript, we refer to them as “open fractures”. We organized the current study around these two goals.

Since BCF cores are scanned by the ImaGeo system, the cores can be examined by image processing techniques^21,33^. Recently, CNNs have proven to be promising tools for the recognition of geological phenomena^39^. Our goal was to construct a perceptive CNN model capable of recognizing open BCF fractures in scanned core samples.

We examined the performance of Mask R‑CNN models^40^ with varying parameters to solve this task. To assess performance, we needed to measure sensitivity (recall) and precision as well as the balance between these two metrics in the F1 score^41^. Given the purpose of the application, we needed to quantify the error statistics of CNN-derived dip dataset performance. Our assessment covered these details, providing insight into the model performance in terms of impeding factors such as dense fracture zones^19^ or technological fractures^14,22,34^. We found this kind of detailed inspection necessary to present the results in a realistic way.

## Material and methods

### CNN

Our study relied on CNN technology, the driving process behind the recently proposed object detection (OD) models^42^. The architecture of a CNN contains complementary parts compared to a plain neural network, as a CNN is designed to perform preliminary feature extraction, transform this information, and pass it on as a compatible input for the network^43,44^. In essence, the input neuron layer accommodates dimensionally reduced “feature maps” created by the convolutional and pooling layers in those preliminary parts of the architecture^45^. This structure is behind both the acquisition of required knowledge and the actual execution of the detection process^43,46^.

### Object detection with Mask R‑CNN

OD essentially marks the location of each recognized object with its own bounding box, providing us with identification and enclosing geometries^40^ that are necessary for dip retrieval. Several models and their subversions exist for OD, and they can be divided into the two main categories of “one-stage” (e.g., YOLO and the single-shot detector) or “two-stage detectors” (e.g., R‑CNN, Faster R‑CNN, Mask R‑CNN) in a simplified manner^47^. “Region proposals” were initiatives of Ren et al. (2017), who described them together with their newly presented Faster R‑CNN model. Their use enables effective detection in cases where the presence of objects has been previously perceived and indicated^48^. Utilizing this step, “two-stage detectors” are considered more refined methods regarding the spatial accuracy of delineation, while their drawback concerns the intricacy of favorably optimizing the components^47^.

Among them, Mask R‑CNN is a well-established choice in terms of the BCF fracture problem because of its “multitask training” and the associated benefits compared to, e.g., Faster R‑CNN^40^.

OD enables us to extract the dip angles without the need for object masks, but Mask R‑CNN is even beneficial for the solely needed bounding boxes due to the way it performs three types of loss calculations during error backpropagation^40,49^. Since bounding boxes were the basis for our dip automation strategy, this is an important aspect that eventually led us to utilize Mask R‑CNN. In the literature, Mask R‑CNN has also been applied to problems such as pavement crack detection^50^.

The preliminary tests we conducted before choosing Mask R‑CNN are summarized in Lovász et al.^26^.

### Preparation of training samples and data for evaluation

Our base material for both training and model testing included scanned BCF core samples. They were collected from.

- Ibafa–4 (Ib–4; 214.39 m for training, 11.13 + 1.60 m for evaluation),
- Bakonya–5 (Bak–5; 30.00 m for training, not used for evaluation),
- Boda–6 (Bo–6; not used for training, 18.43 m for evaluation).

All datasets described in the article are owned by the PURAM and were provided to us for research purposes. Originally, the scanned borehole imagery was stored as separate image files, where the depth was indicated by the file name only. To make the training procedure easier, all individual images were organized into a single image mosaic in ArcGIS Pro (ver.: 2.8.4; Environmental System Research Inc., Redlands, CA, USA), with the supplementary use of PURAM core scanning logs, core documentation, and Python scripts to automate the correction process. After this step, we were able to handle the images of a particular borehole as a single, continuous raster, alleviating the data management issue and the training procedure.

To train our Mask R‑CNN model, we selected two mosaics made from the scanned images of Ib–4 and Bak–5. On these mosaics, we digitized 367 open fractures according to their precise segmentations. In our research, we utilized Mask R‑CNN for OD because this model is not only applicable for instance segmentation but also for finer object delineation after learning on segmented data^40^. We utilized ArcGIS Pro to digitize detailed fracture geometries as single- or multipart polygons. The sampling distance of the original Ib–4 and Bak–5 mosaics was 0.075 mm^29,33^. With this resolution, the input imagery contained c. 3 500 pixels around the perimeter of the cylindrical core samples^22,29,33^.

Mask R‑CNN, similar to other CNNs, processes images in tiles. Mask R‑CNN effectively adapts to its original ResNet backbone tile size, which is 224 × 224 pixels^40,51^. Therefore, we had two choices: either use the original resolution and cut the training images from into 224 × 224 tiles (1) or resample the whole raster mosaic to fit this 224 × 224 tile size (2).

1. In the first case, we could preserve and maintain the fine details that were visible on the core sample, but the CNN would not be able to see the fractures in their geological context. Without this informative context, as Yamashita et al. (2018) wrote, deficient feature extraction would take place due to this incorrect CNN point of view and would likely be degraded to the plainer level of feature extraction. Finding informative contexts for CNNs is crucial^52^.
  Hence, sticking to the first case is disadvantageous even for the simplest fractures and prohibits distinguishing features with technological origins from tectonic processes. Most commonly, the fractures cross the whole core diagonally, so they are split into several individual tiles with no supplementary information for the training procedure. This information, however, is essential for each fracture, as their automated extraction requirement applies to their entire arc.
  For fractures with multipart arcs/coexistence between the main process and subprocesses^22,35^ or with any complexity at all regarding relations and continuity, this information is indispensable. Consequently, the CNN output would fail the fundamental goal if the 224 × 224 training chips were cut multiple times from the original mosaic resolution.
2. In the second case–after resampling–the whole result of a geological process is visible on a single tile in its context. Training in this manner may enable Mask R‑CNN to recognize fractures in their contexts. In this case, the sampling distance degrades to 0.96 mm.
  Based on these considerations, we chose the second option even if we lost some details of the scanned core.

We used a 112 × 112-pixel stride to make the recognition process less dependent on the positions of fractures in the exported tiles^53^.

GIS mosaics and resampled images are not suitable for common deep learning tools, so we had to export them as R‑CNN masks, which are the native inputs of the Mask R‑CNN training procedure^54^. The export operation was performed by the ArcGIS “Export Training Samples for Deep Learning” geoprocessing tool. The output was based on the digitized polygons and on the resampled raster mosaic.

### Training

In terms of deep learning, this amount of data (367 samples) is rather limited yet can effectively be used through “transfer learning” with the available backbone architectures^55^. Among the many groups of available feature extractor bases, “residual neural networks” (the ResNet family, introduced by He et al.) have enhanced the utilized training strategies with their “shortcut connections” as they banished the “vanishing gradient” problem that often occurs during backpropagation^51^.

The utilized ResNet‑18, ResNet‑34, ResNet‑50, ResNet‑101, and ResNet‑152 are deep residual network subversions with an increasing number of hidden layers^51^.

We carried out tests with all these variants. Backbone comparisons are frequent in deep learning studies^56,57^. Criticism regarding networks that are too deep, or regarding the consideration of depth as a sole aspect of performance has appeared in the work of^57,74^. The relationship between the network depth and performance may vary e.g., by the tackled task^56,57,74^. More depth often, but not decisively seems practical for tasks with higher complicacy^57,74^.

To train, apply and evaluate the models, we used ArcGIS Pro’s deep learning framework (arcgis.learn module, version 1.8.5,^54^). Technically, this framework is a collection of convenience functions built on top of the standard open-source FastAI, PyTorch, and TensorFlow packages and their partially overlapping dependencies^58^.

To train the models, we used the “Train Deep Learning Model” geoprocessing tool. For each training session, a maximum of 20 epochs were allowed with the “early stopping” function of the tool enabled, and the batch size was 4^59^. This size was necessary due to hardware limitations^60^.

There is a scientific approach in which a system trained in subtle batches is presumably less prone to overfitting due to the presence of noise in such a small training batch^61^. Each model was consistently set to the default batch size of 4. Each model structure was “unfrozen” to enable automatic parameter adjustment on every layer^54^.

During training, the chosen optimizer algorithms minimize the loss function (the degree of error) by varying certain model hyperparameters, depending on the approach^62^. The hyperparameters of the network (e.g., learning rate) are subtle but potentially vital details in the ultimate learning process^63^.

ArcGIS Pro’s “Train Deep Learning Model” geoprocessing tool does not offer optimizer selection on its user interface, but this function was identified in the underlying packages^54,58^ that the training tool calls an exact open-source FastAI code, which has adaptive moment estimation (‘Adam’;^64^) as its default^65,66^. Adam is one of the so-called “adaptive optimizers”, achieving finer networks by specializing a particular learning rate for each parameter^64^.

Adam recently became popular overall in artificial neural network (ANN) applications, but it is not guaranteed to excel in all tasks without exception. In several cases, its generalization ability was found to be weaker than that of nonadaptive stochastic gradient methods, such as stochastic gradient descent (SGD) with momentum^67^.

To conduct an experimental validation, we carried out several training tests with the use of ArcGIS Pro’s default Adam optimizer, and we also modified the optimizer on the script interface to SGD combined with momentum within the best-performing model variations. The latter was done through a Python script using the arcgis.learn library^54^.

Finally, we trained 5 + 1 models. First, we made five models based on ResNet‑18, ResNet‑34 ResNet‑50, ResNet‑101, and ResNet‑152 with the Adam optimizer; the model with the most appropriate backbone was selected, but its optimizer was changed to SGD combined with momentum.

Two models (ResNet‑18 and ResNet‑34) were later rejected due to their poor performance.

### Detection

When each model was fully trained to estimate its capabilities and quality, we ran the “Detect Object Using Deep Learning” geoprocessing tool on our evaluation datasets with a detection tile size of 224 × 224 pixels. To match ResNet’s default tile size, the test datasets were also downsampled and cut into tiles with sizes of 224 × 224 pixels, allowing each deep learning model to extract open fractures along the circumference of the cylinder of the given core sample. Mask R‑CNN has the capability to produce detailed polygons as outputs (for instance, segmentation results) and to produce bounding boxes and work as an object detector tool^40^. We chose the latter, enabling bounding box generation in the tool to serve our dip calculation purposes. The confidence level was 0.5^54^.

### Evaluation and selection of core sections

Validation datasets are primarily subjects of the training process, designed to aid the hyperparameter adjustment process within the neural network^68^, and they are not applicable for proper evaluations, which must be carried out independently^68–70^.

To comply with the independent evaluation paradigm, we digitized evaluation polygons (ground-truth polygons) on the evaluation sections of the mosaics of Ib–4 and Bo–6 (prepared as described in point section “Preparation of training samples and data for evaluation”). The ground-truth polygons and the sections separated for evaluation were strictly distinguished from the training area and polygons: neither instancewise nor spatial overlapping was enabled. For evaluation, we digitized 156 fractures altogether from Ib–4 and Bo–6. Subsequently, these detailed polygons were generalized to bounding boxes to match the outputs of the evaluation runs.

#### Core sections selected for model evaluation

The two evaluation sections from Ib–4 contained 74 (44 and 30) fractures, while the Bo–6 section included 82 ground-truth fractures (156 in total).

Among the scanned core materials tested, the best quality came from Ib–4. This enabled us to test a wide range of fracture phenomena, as discussed in the section “Introduction”.

To carry out a detailed evaluation and to obtain partial results supporting our model construction, we found it important to start evaluating sections from this core mosaic.

The Ib–4 core areas selected for model assessment did not participate in the training process, ensuring that our testing ran on data that were strictly unknown to the models. Selecting these areas, we specifically split the data into a moderately difficult section (Section 1) and a section with higher complexity (Section 2) for the Ib–4 mosaic.

- **Section 1** (11.13 m) represented the vast majority of BCF characteristics. This section predominantly consisted of single fractures with slight difficulty due to several hardly noticeable fractures. Only a very few minor intersections were present as ground truths. Samples from Section 1 can be seen in Figs. 4 and 5.
- **Section 2** (1.64 m) represented many multipart and/or intersecting fractures that are challenging to comprehend even through a profound visual analysis executed by a trained geologist. It was mostly composed of dense fracture zones. It is important to note that this level of critically complex fault interlacement was not typical in the training database (since we tried to adjust their proportions to the most common levels for the BCF). Samples from Section 2 be seen in Figs. 6 and 7.

**Figure 3:**
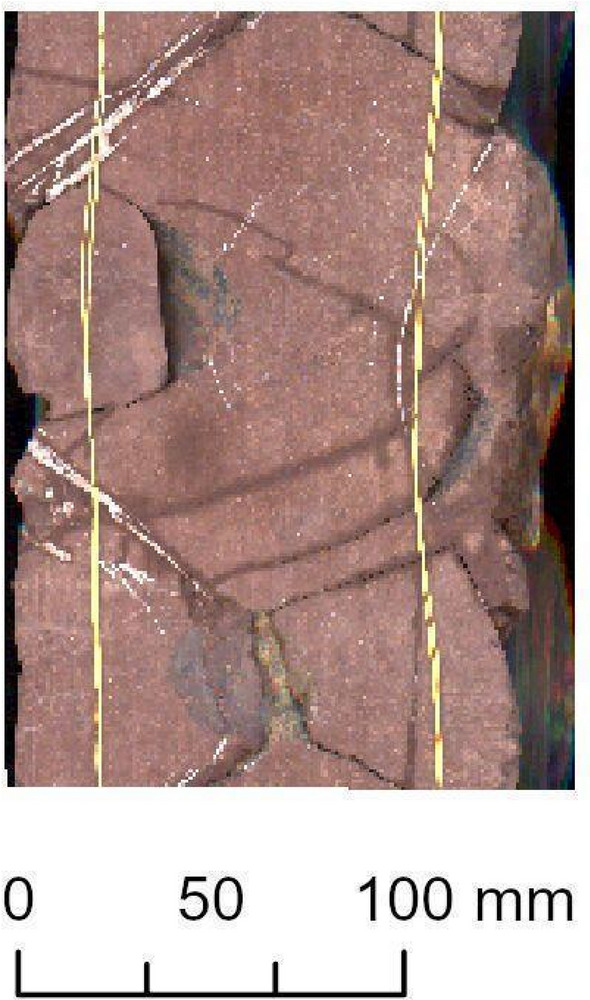
Examples of core samples excluded from the Bo–6 assessment due to multiple technological complications. Note: The image quality was determined by resampling (224 × 224 pixels), as described in the study. The samples are based on PURAM data.

We evaluated the model on both parts separately and aggregated them into one section to determine the overall performance. The highest-performing Mask R‑CNN model was evaluated on the scanned core of the additional Bo–6 borehole. It is relevant to note that the condition of Bo–6’s core material was unusually fragmented. In the case of the Ib–4, the scanned core was almost completely coherent in its full extent (as would be ideal for any borehole). Consequently, while testing on Ib–4, we did not encounter notable technical hurdles. Completing the evaluation on Ib–4 Section 2, we only excluded a very subtle, 8.4-cm section from the original 1.64-m length that was deemed uncertain for the ground truth due to its geological interpretation difficulty. The available Bo–6 scanned core, however, required preselection prior to running our deep learning models on it, achieving a circa 18.43-m long raster (spatial extent). A few parts further had to be excluded from the evaluation (e.g., Fig. 3.). This was most commonly due to their levels of fragmentation and interruption. The samples in which we saw a majority of difficult-to-interpret fractures due to technological reasons were also excluded^14,22,34^. Combined with the abovementioned conditions, these fractures proved very difficult to interpret even visually.

Fracture lines along joint surfaces^27^ were also not included in our target. They were present in large numbers of the scanned core samples of Bo–6 and were excluded from the performance survey because, for the reason described above, we did not register them in the ground-truth feature class.

### Evaluating metrics for the Mask R‑CNN model results

Each model composition was evaluated on the selected sections through ArcGIS Pro’s “Compute Accuracy for Deep Learning” geoprocessing tool for comparing each output to the previously registered ground-truth (bounding box) features. The calculated recall, precision, and F1 score values were all vital aspects of our models. The “intersection over union” (IoU) threshold was set to the generally accepted level of 0.5^54,71^.

### Retrieving dip values and evaluating their validity

After obtaining the bounding box output of the Mask R‑CNN, we could calculate dip values from it. For each box, either the coordinates of all the corner points or at least the side ratios needed to match the relevant properties of the ground-truth bounding box. To prepare, we first extracted each individual dip value for the ground-truth fractures and model-generated output fractures alike via Python scripting (see Supplementary Information 1) relying on the inverse tangent, where individual, two-dimensional extents were previously calculated with ArcGIS Pro’s “Add Geometry Attributes” geoprocessing tool.

In this task (unlike in the detection task), only fractures with at least half the circumference of the core could be considered. In many cases, some parts of fracture lines were filled^21,22^. In these instances, our model potentially would delineate only the open (and within-core) part of a composite fracture and could return misleading dip information about the tectonic process. However, if their open part reached half of the sinus wave, the extracted dip value would remain authentic. We did not venture to carry out dip automation and evaluation on Bo–6 due to the previously described levels of fragmentation and interruption that resulted in both peaks of many fracture waves being outside of the available scanned image.

Having obtained the correct dip values, we joined the ground-truth feature class relating to each CNN bounding box feature class in a relational database, through which any further comparison or statistical calculation could be performed. The coding step could not rely on the assumption that the sequence of joining elements (from the output and ground-truth feature class elements) was sorted according to the order of indexing (because of the presence of false-positives/false-negatives and the subjective order of digitizing ground truth). The related elements of key-value pairs (ground truth and CNN output) were connected by careful manual selection. The ‘dip’ columns of each CNN output feature class were joined to the ground-truth attribute table. Error calculations and further statistical analyses of key-value pairs were only considered in cases where the comparison satisfied the minimum IoU value (0.5) set during the relevant object detection evaluation. This was ensured through ArcGIS’ ArcPy^72^ (Supplementary Information 2) prior to calculating the error (expressed in absolute values) statistics regarding dipping datasets (Table 1).

**Table 1:**
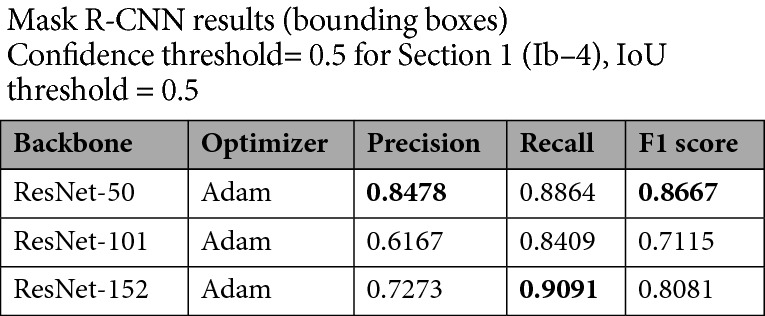
Results obtained on Ib–4 Section 1 with the default Adam optimizer.

Best values are in bold.

## Results

### Object detection results obtained on Ib–4 Section 1

**Figure 4:**
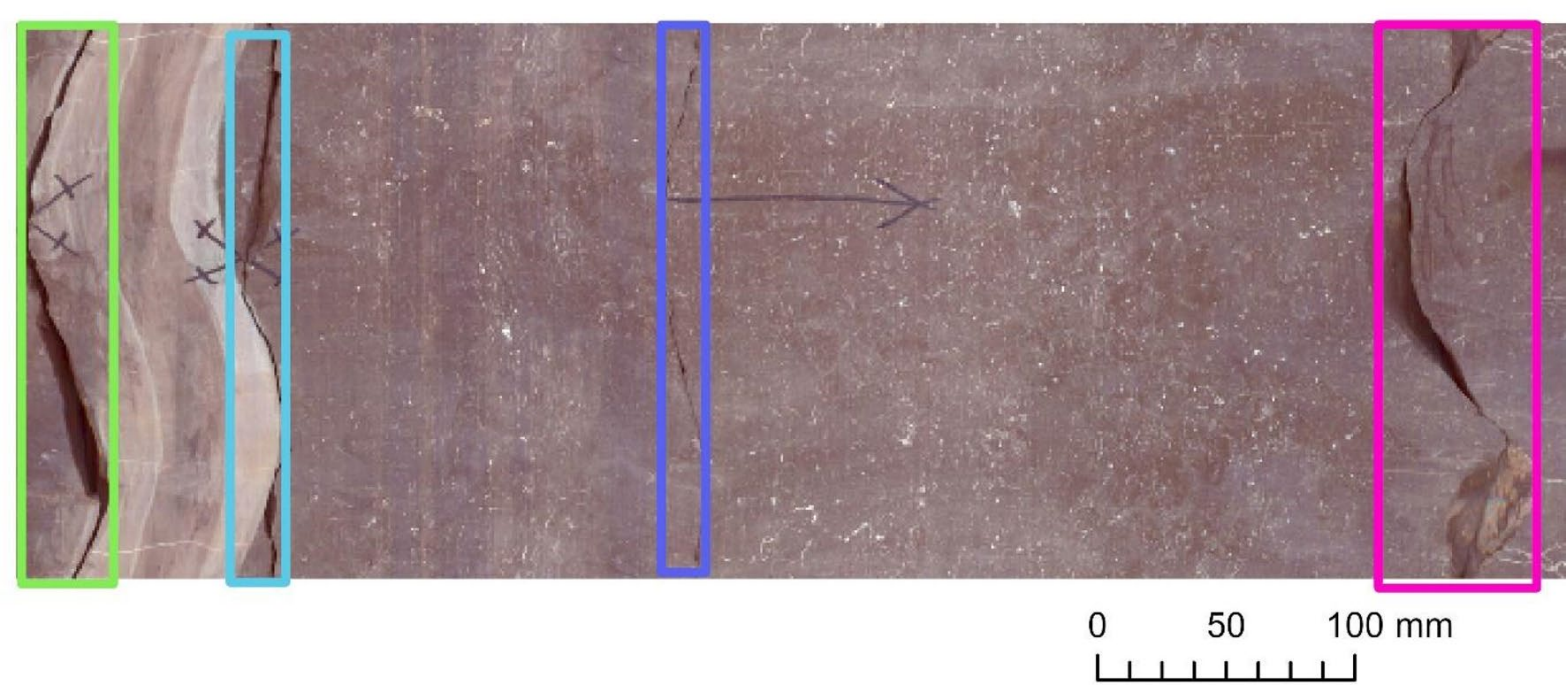
Examples of detected fractures (with the ResNet‑50 backbone) in Section 1. Note: Image quality depends on necessary resampling (224 × 224 pixels), as described in the text. Based on PURAM data.

**Figure 5:**
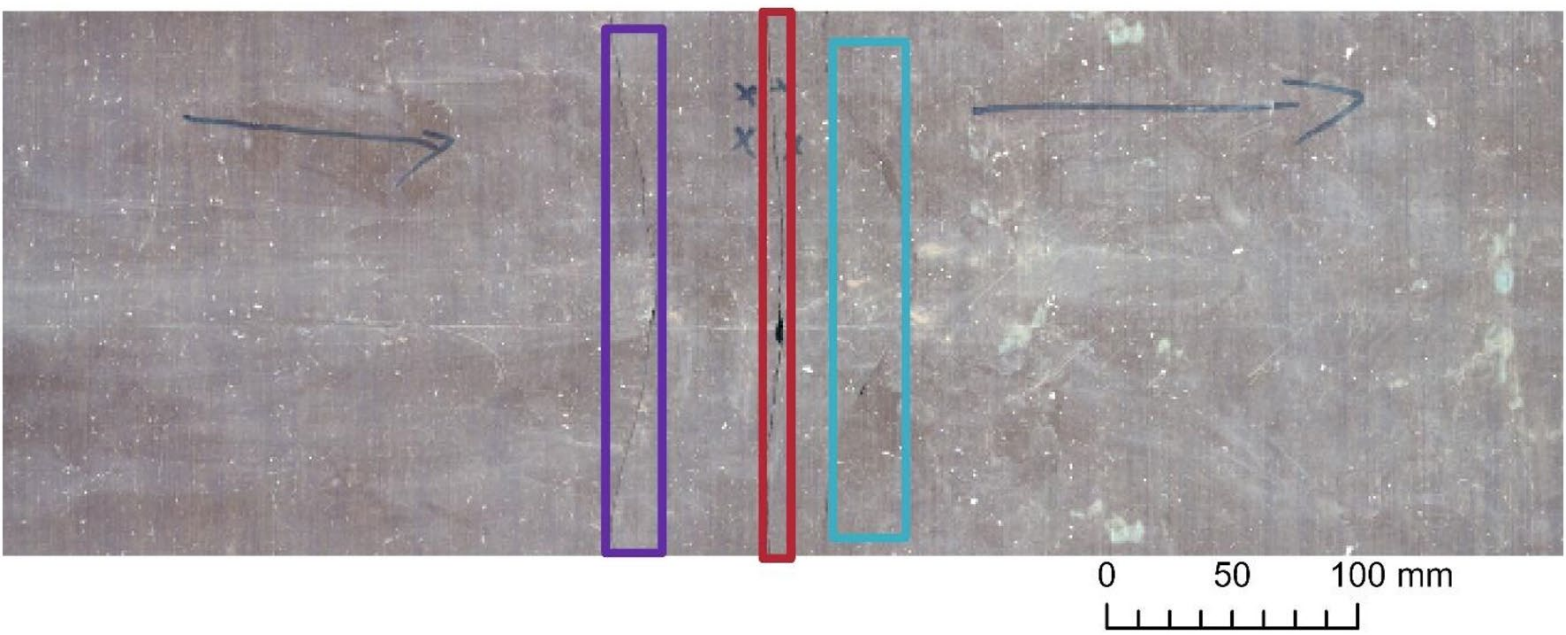
Examples of detected fractures that are barely visible to the naked eye (with ResNet‑152) in Section 1. Note: Image quality depends on necessary resampling (224 × 224 pixels), as described in the text. Based on PURAM data.

Based on the testing performed on the moderately complex Section 1, all three examined constructions perceived data very sufficiently (Fig. 4) even for the finest fractures (Fig. 5). A competition was formed between the shallowest (ResNet‑50) and deepest (ResNet‑152) backbones. Although the result obtained with ResNet‑50 was the most precise and most balanced result (but clearly deteriorating when optimized with SGD instead of Adam – Table 2), the circa 91% sensitivity of ResNet‑152 was remarkable (Table 1).

**Table 2:**
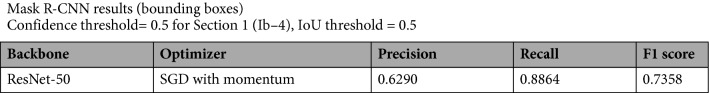
Partial results obtained on Ib–4 Section 1 with the optimizer changed to stochastic gradient descent (SGD) supplemented with momentum.

When inspecting the lower precision and higher recall rate demonstrated by ResNet‑152 (when compared to ResNet‑50), we observed that the higher number of false-positives could be attributed to two factors. Since the sine waves along the lines of joint surfaces were not related to our goal (nor were they related to our training data), they were not registered as elements of the ground-truth feature class. However, our model (exhibiting high sensitivity) with ResNet‑152 recognized two of these locations (thus registering them as false-positives). However, this marginal quantity barely contributed to the lower precision (improving only to ~ 0.7547 when joint surface recognition was excluded). More often, this was due to multiple delineations of a fracture (with varying bounding box sizes) in several cases.

### Results obtained on Ib–4 Section 2

**Figure 6:**
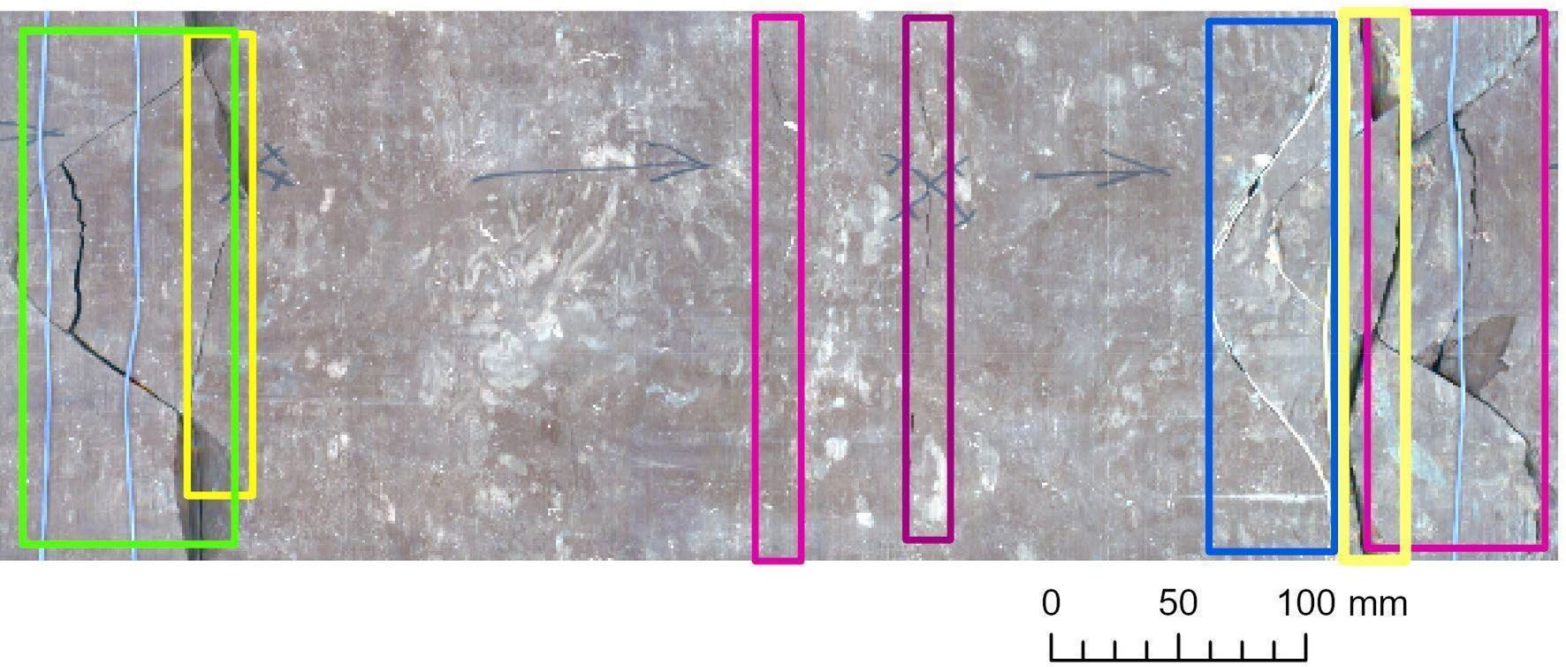
Examples of the Mask R‑CNN results obtained on Section 2. Note: Image quality depends on necessary resampling (224 × 224 pixels), as described in the text. Based on PURAM data.

**Figure 7:**
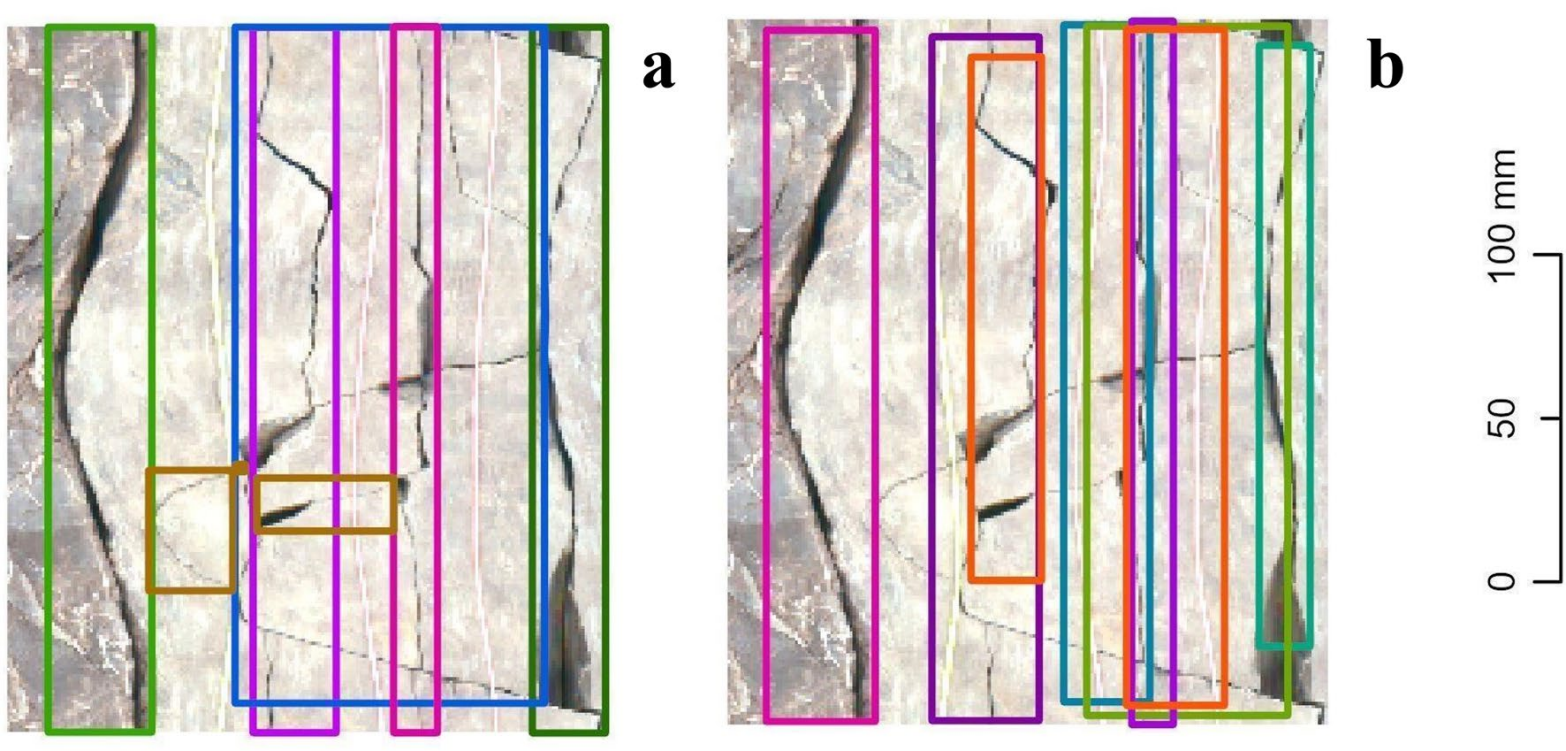
Example results obtained on Section 2 of Ib–4. Note: (**a**) Ground truth; (**b**) Mask R‑CNN. Note: Image quality depends on necessary resampling (224 × 224 pixels), as described in the text. Based on PURAM data.

Applying Mask R‑CNN to Section 2 was more difficult, as the main focus was on fracture zones (with fractures characterized by intricate intersections and many subtle subprocesses). Accordingly, the achieved performance was less satisfactory (Table 3). While ResNet‑152 was found to be highly sensitive in the previous section, quite the opposite occurred in the presence of more intricate fracture shapes. However, ResNet‑50 performed well in terms of all three metrics, and at this level, it had the highest recall rate. To a certain extent, this model built on ResNet‑50 perceived fractures with any thickness level, whether they were single or intersecting. As Fig. 6 demonstrates, common inaccuracy was likely to be found in the IoU value, e.g., when the filled gaps of an open fracture were not included in the bounding box. This is because our model was trained on a dataset that consisted exclusively of open fractures. Dominantly filled fractures can also occur as false-positives, although for some future uses of the model, this will not always be accounted for as an error. We can observe the relatively good suitability of ResNet‑50 with respect to all three metrics, considering that the features Mask R‑CNN marked here were equivocal (perceived minor subprocesses are potentially technological fractures) and visually challenging even to experts with years of geological experience (Fig. 7). This time, optimizing the network with SGD improved the precision but only at the cost of recall and resulted in a very low F1 score. (Table 4.)

**Table 3:**
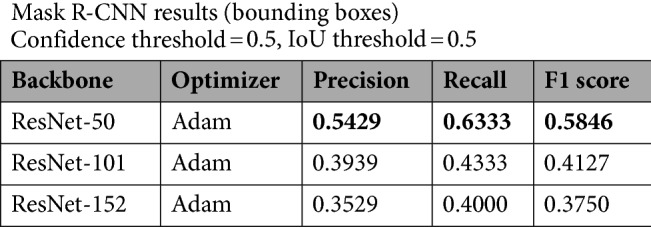
Results obtained on Ib–4 Section 2 with the default Adam optimizer.

Best values are in bold.

**Table 4:**
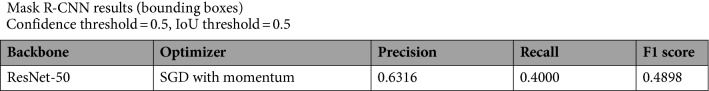
Partial results obtained on Ib–4 Section 2 with the optimizer changed to SGD supplemented with momentum.

### Object detection results obtained on the combined sections of Ib–4

The combination of Sections 1 and 2 represents a wide range of fracture types and complexity levels, as well as some contributing factors that may have resulted from almost inevitable core damage. Through the combination, we wanted to ensure that we eliminated bias from the model performance results and that we highlighted many realistic impeding factors. The results demonstrated in Table 5 are therefore strongly realistic. On this basis, training and applying Mask-RCNN in the way described above is a powerful and reliable solution if utilized with ResNet‑50 and the Adam optimizer. It can be applied very effectively to BCF fractures with the supplementation of manual checking and (if necessary) correction/completion.

**Table 5:**
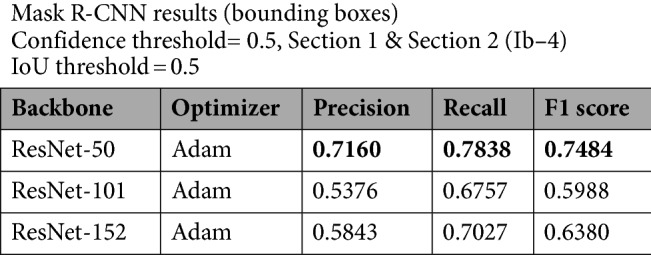
Results obtained on the combined Sections 1 and 2 of Ib–4 with the default Adam optimizer.

Best values are in bold.

### Object detection and accuracy measurement on Bo–6

**Figure 8:**
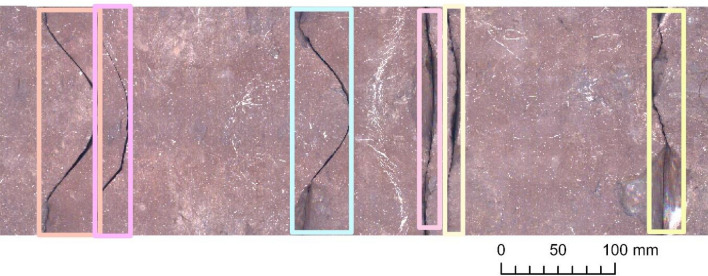
Examples of detected fractures obtained on Bo–6. Note: Image quality depends on necessary resampling (224 × 224 pixels), as described in the text. Based on PURAM data.

**Figure 9:**
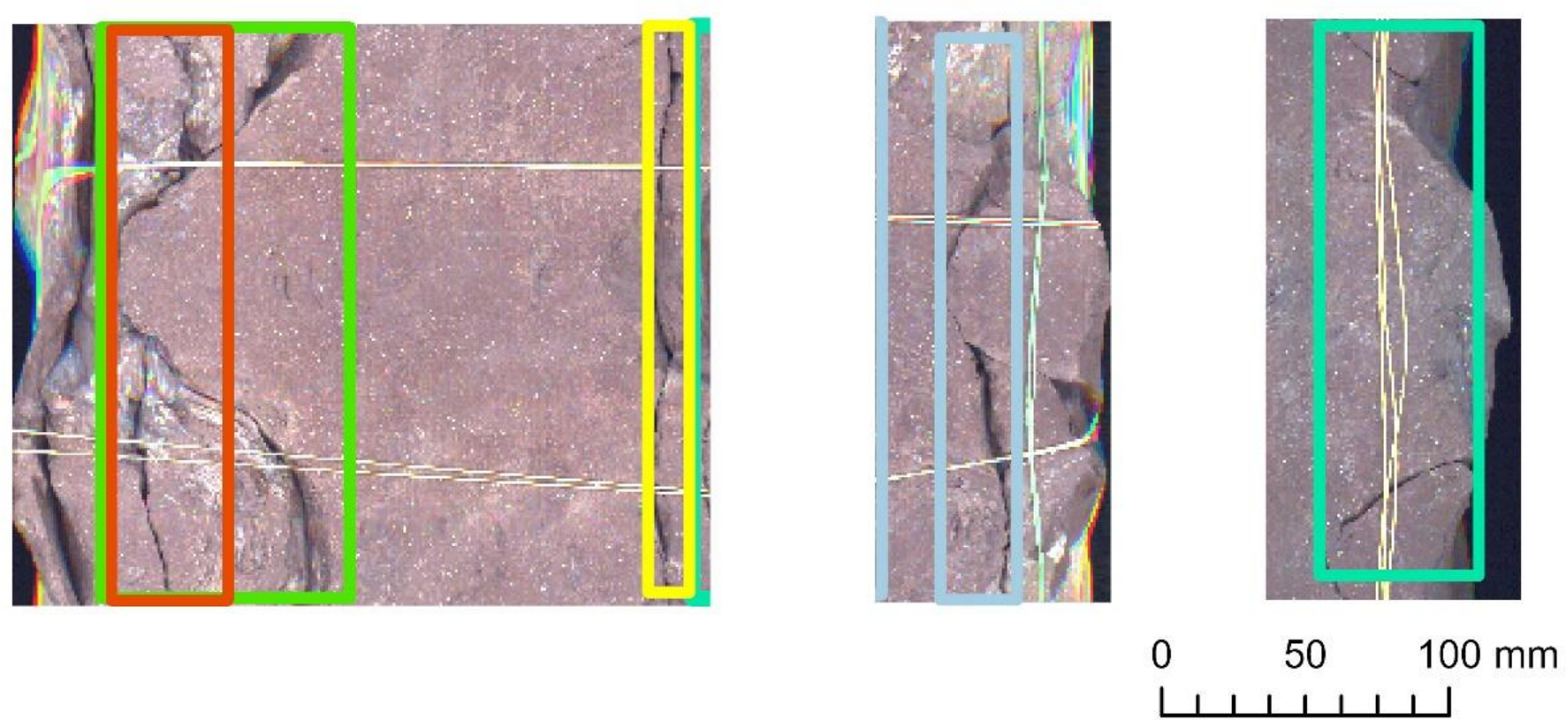
Examples of detected fractures obtained on Bo–6. Note: Image quality depends on necessary resampling (224 × 224 pixels), as described in the text. Based on PURAM data.

**Figure 10:**
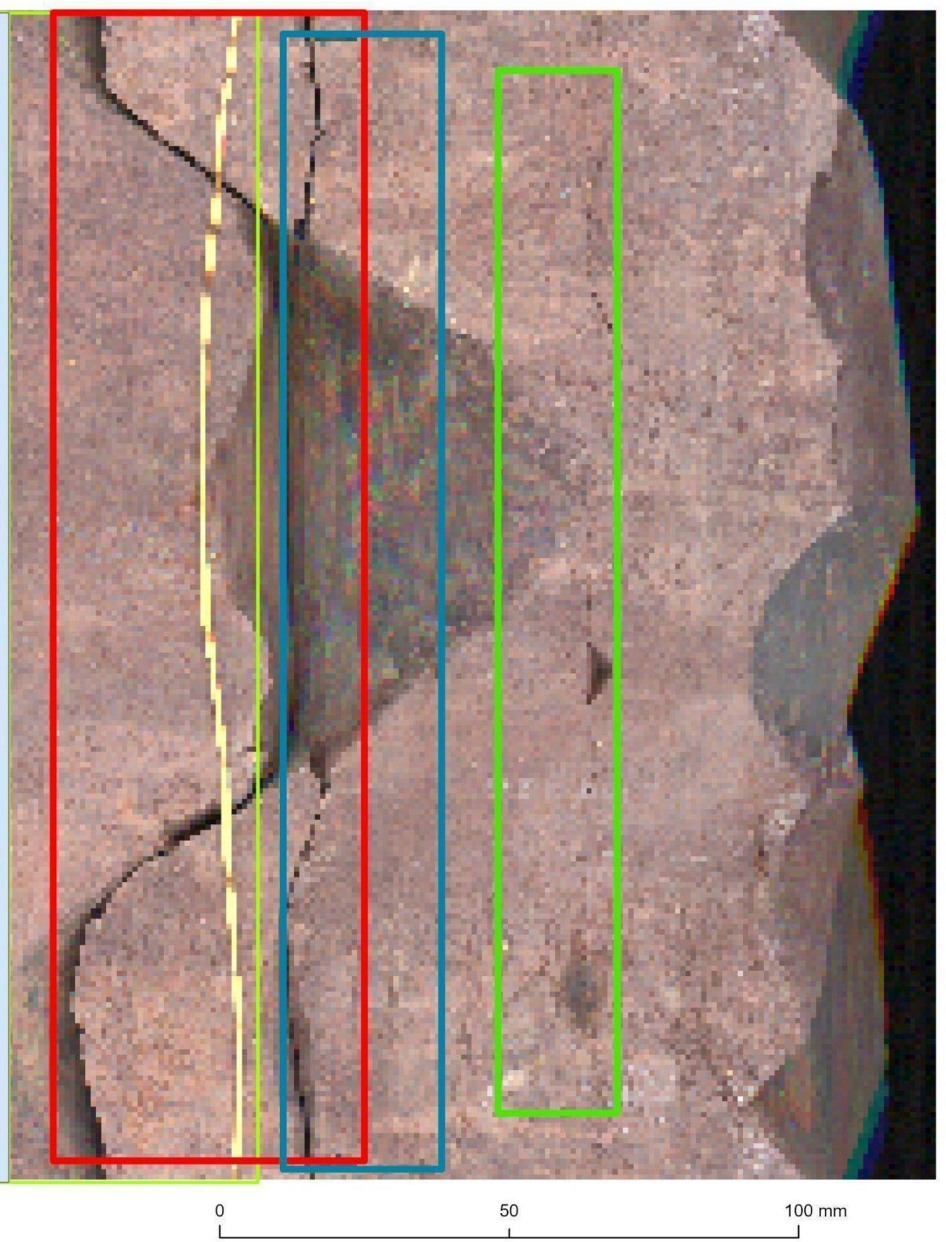
Examples of detected fractures obtained on Bo–6. Note: Image quality depends on necessary resampling (224 × 224 pixels), as described in the text. Based on PURAM data.

We ran the previously customized Mask R‑CNN model version on the scanned image mosaic of the Bo–6 borehole (Table 6) for evaluation purposes. As discussed in 3.1, the performance was strong up to a certain level of fracture complexity (Fig. 8). The specifically tailored training and detection method seemed to succeed in recognizing the cohesive arcs of multipart fractures (Fig. 9), perceiving hairline fractures and detecting intersecting fractures (Fig. 10) in some places. However, the latter task was only partially successful, which is also demonstrated by the numbers of false-positives and false-negatives, and the model indicators.

**Table 6:**
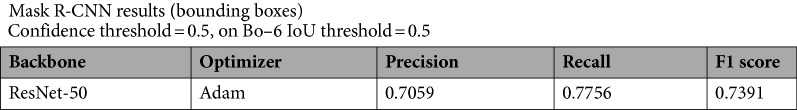
Detection results obtained with the use of the Adam optimizer and ResNet‑50 backbone, which were ran on Bo–6.

### Evaluating the dipping dataset derived from Ib–4

**Figure 11:**
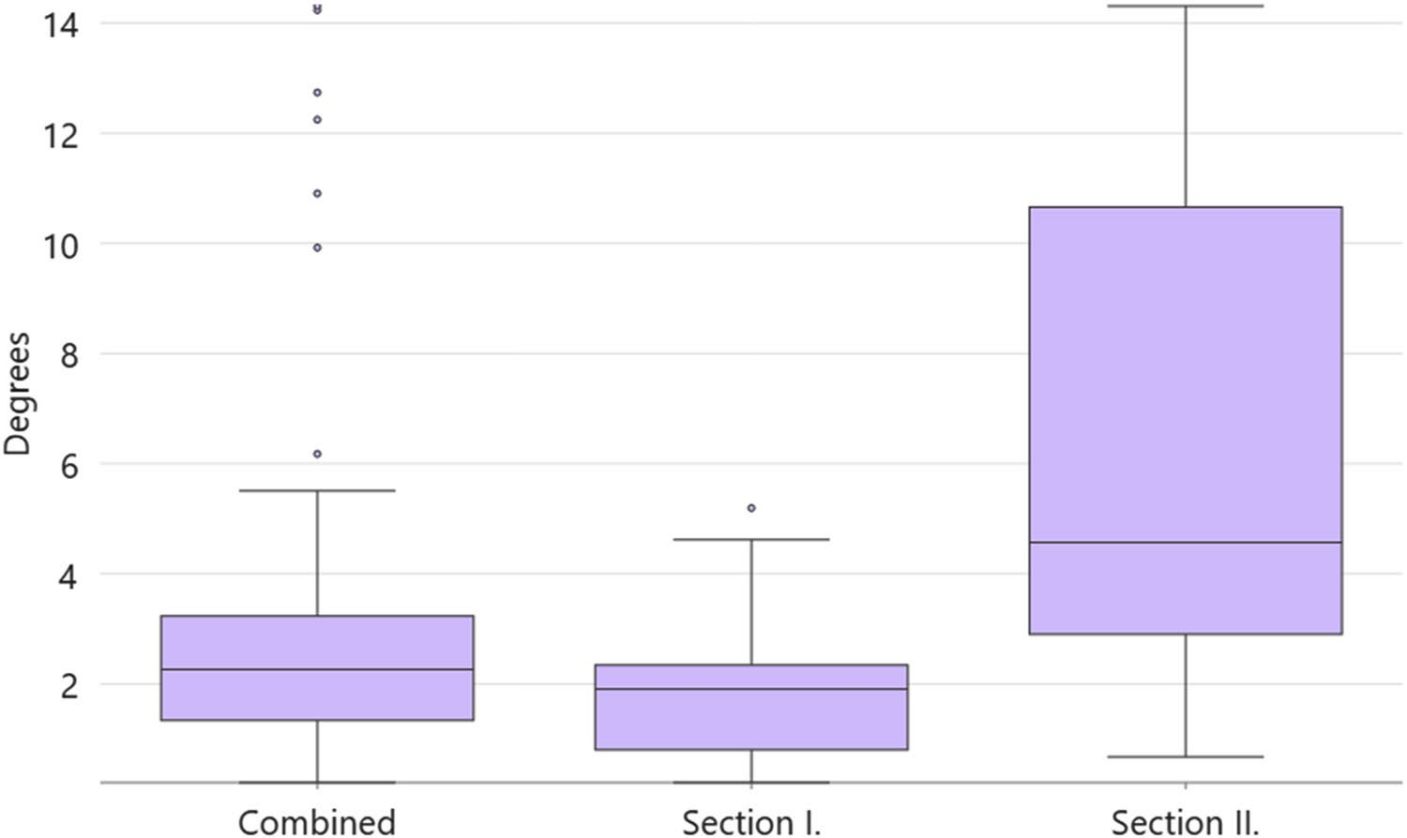
Error boxplot of the CNN-derived dipping dataset for the combined section of Ib–4. Based on PURAM data.

Another goal was ensuring that the generated bounding boxes of the CNN’s output represented all tectonic processes in a geologically authentic way so that they could be generally used for automated dip calculations. During the manual recording procedure, values within 5 degrees of error could genuinely occur due to manual inaccuracy and should be considered within the acceptable limit^22^. For the Mask R‑CNN model supplemented with subtle postprocessing coding, the output seemed to outperform those of the abovementioned models by not reaching the median errors of 1.9° for Section 1 and 4.6° for Section 2 (Table 7 and Fig. 11). It is important to highlight that Section 1, which represents the simpler fracture phenomena predominant in the core, yielded lower errors.

**Table 7:** Evaluation results obtained the CNN-derived dipping dataset of Ib–4.

	Mean error [°]	Minimum error [°]	Maximum error [°]	Standard deviation of error [°]	Median of error [°]
Section 1	1.8	0.2	5.2	1.2	1.9
Section 2	6.4	0.7	14.3	4.6	4.6
Combined	3.3	0.2	14.3	3.5	2.3

Considering this, the automated dip extraction strategy from Mask R‑CNN fulfilled our expectations.

### Known limitations

During our research, we made every effort to prevent inconsistencies or misleading results/information. Even with these safeguards, conducting applications with the utmost care must also be balanced with considering the boundaries/elements of the uncertainties rooted in core processing, borehole documentation and geological interpretation. Among the many inherent uncertainties, there can be cases when the current condition of a visible fracture exhibits forms that are slightly modified from the original geological process.

In the numerical evaluation of the model variants presented, any fine detail omitted by Mask R‑CNN was attributed as a limitation/deficiency of the model because we insisted on setting the level of expectation remarkably higher than PURAM’s guideline for (labor-intensive) manual core documentation.

Our dip calculation in the presented workflow did not cover drilling inclination; in the case of a deviated well, the deviation angle of the borehole needs to be subtracted from the calculated dips to obtain the real value.

While the evaluation exhibited (in some respects) unexpectedly high performance, the results referred to certain core samples. Beyond what is described in the evaluation, it is unknown to what extent this performance achieved by the presented model can be generalized^73^. The fragmentation of a core strongly influences applicability. Moreover, the final model proved only partially successful in terms of the correct extraction and separation of intersecting and/or multipart-type fractures, and its performance seemed to be sounder for single fractures. Since (as the literature review in the introduction has suggested) data on both open and filled fractures alike are expected for comprehensive modeling, it is necessary to train the presented model further to extend its capability to handle both types of fractures.

## Conclusions

We thoroughly assessed the behavior, possibilities, and limitations of an object detection and GIS processing method for different cases of BCF fractures. The ultimate aim of this study was to support data provision. The presented geospatial workflow can automatically extract certain basic data that are relevant to stress field-related or hydrogeological studies. With additional transfer learning, the capacity of the model can be expanded to provide a more comprehensive dataset for important surveys supporting DGR planning.

## Supplementary Information

Supplementary Information 1.

Supplementary Information 2.

## Data Availability

The authors do not own any of the datasets used in this research. Every dataset is the property of the Public Limited Company of Radioactive Waste Management (PURAM). The datasets are restricted and available with permission only. Contact titkarsag@rhk.hu at the PURAM to request permission. We request the same if you require any of the referenced manuscripts regarding the BCF.

## References

[1] Witherspoon, P. A., Bodvarsson, G. S. (eds). *Geological Challenges in Radioactive Waste isolation: Third worldwide review.* Report number: LBNL-49767. 335 p. (Ernest Orlando Lawrence Berkeley National Laboratory, Berkeley, CA [United States], 2001).

[2] Nuclear Energy Agency. Management and Disposal of High-Level Radioactive Waste: Global Progress and Solutions. OECD NEA No. 7532, 51 p. *OECD Publishing, Paris* Available: https://www.oecd-nea.org/upload/docs/application/pdf/2020-07/7532-dgr-geological-disposal-radioactive-waste.pdf (2020). Accessed 23 January 2023.

[3] Nős B (2020). Needs of countries with longer timescale for deep geological repository implementation. EPJ Nucl. Sci. Technol..

[4] Warner P. J. United States of America Activities Relative to the International Atomic Energy Agency (IAE) Initiative: Records Management for Deep Geological Repositories [conference paper—1997 Waste Management Conference, Tucson Arizona] 18 p. https://www.osti.gov/biblio/451245 (1997). Accessed 22 February 2022.

[5] Apted, M., Ahn, J. (eds.) Geological Repository Systems for Safe Disposal of Spent Nuclear Fuels and Radioactive Waste. 802 p. ISBN: 9780081006429 (Woodhead Publishing Series in Energy, 2017).

[6] Lázár, K., & Máthé, Z. Claystone as a Potential Host Rock for Nuclear Waste Storage. In: Valaskova, M., Martynkova, G. S. (eds.) *Clay Minerals in Nature - Their Characterization, Modification and Application*. Rijeka, Croatia: InTech (2012) pp. 55–80, DOI: 10.5772/48123 (2012).

[7] Baksay, A. et al. Current Status of Geological Disposal Projects in Hungary. Chapter 10 in: Faybishenko, B., Birkholzer, J., Sassani, D. and Swift, P. International Approaches for Nuclear Waste Disposal in Geological Formations: Geological Challenges in Radioactive Waste Isolation — Fifth Worldwide Review. LBLN-1006984 https://www.osti.gov/servlets/purl/1353043 (Berkeley, CA, United States, 2016). DOI: 10.2172/1353043

[8] Németh T, Máthé Z, Pekker P, Dódony I, Kovács-Kis V, Sipos P, Cora I, Kovács I (2016). Clay mineralogy of the Boda Claystone Formation (Mecsek Mts., SW Hungary). Open Geosci..

[9] PURAM [RHK]. Ismét munkában a fúrógép. https://rhk.hu/hirek/2020/10/30/ismet-munkaban-a-furogep-1 (2020). Accessed 05 March 2022.

[10] Konrád Gy, Sebe K, Halász A, Babinszki E (2010). Sedimentology of a Permian Playa Lake: the Boda Claystone Formation, Hungary. Geologos.

[11] Kovács L, Hámos G, Csicsák J (2000). Actual state of the site characterisation programme of the Boda Siltstone Formation. Bull. Hung. Geol. Soc..

[12] Varga A, Raucsik B, Szakmány G, Máté Z (2006). A Bodai Aleurolit Formáció törmelékes kőzettípusainak ásványtani, kőzettani és geokémiai jellemzői. [Mineralogical, petrological and geochemical characteristics of the siliciclastic rock types of Boda Siltstone Formation]. Földtani Közlöny.

[13] Tóth E, Hrabovszki E, Schubert F, M.Tóth T (2022). Lithology-Controlled Hydrodynamic Behaviour of a Fractured Sandstone–Claystone Body in a Radioactive Waste Repository Site, SW Hungary. Appl. Sci..

[14] Hrabovszki E, Tóth E, Raucsik B, Varga A, Schubert F (2017). A BAF–2 fúrás töréses szerkezeti elemeinek mikroszerkezeti és cementációvizsgálata (Bodai Agyagkő Formáció). [Microstructure and cementation analyses on core samples from the BAF–2 well (Boda Claystone Formation, Mecsek Mts)]. Földtani Közlöny.

[15] Fedor F, Máthé Z, Ács P, Koroncz P (2018). New results of Boda Claystone research: Genesis, mineralogy, geochemistry, petrophysics. Geol. Soc. Lond. Spec. Publ..

[16] Hrabovszki E, Tóth E, M. Tóth T, Máthé Z (2020). Potential formation mechanisms of early diagenetic displacive veins in the Permian Boda Claystone Formation. J. Struct. Geol..

[17] PURAM [RHK]. A Bodai Agyagkő Formáció telephelykutatási keretprogramjának engedélykérelme – Közérthető összefoglaló. [Application for permission for the site evaluation framework program of the Bodai Claystone Formation – A comprehensible summary.] 9 p. Retrieved from: http://www.nymtit.hu/docs/2019/rhk_kozertheto_osszefoglalo.pdf (2019). Accessed 22 February 2022.

[18] Mirza, M. T., Ali, S., Khan, A. & Hassan, M. Application of Artificial Intelligence for optimized and cost-effective disposal of radioactive waste. [Conference Abstract]. In *International Conference on Radioactive Waste Management: Solutions for a Sustainable Future [Book of Abstracts]* 165. Available at: https://inis.iaea.org/search/search.aspx?orig_q=RN:53084500 (2021).

[19] Tóth E, Hrabovszki E, Schubert F, M. Tóth T (2022). Discrete fracture network (DFN) modeling of a high-level radioactive waste repository host rock and the effects on its hydrogeological behavior. J. Struct. Geol..

[20] Hungarian Government. 155/2014. (VI. 30.) Korm. rendelet a radioaktív hulladékok átmeneti tárolását vagy végleges elhelyezését biztosító tároló létesítmények biztonsági követelményeiről és az ezzel összefüggő hatósági tevékenységről. [Government Decree on the safety requirements for storage facilities providing temporary storage or disposal of radioactive waste and related official activities – 155/2014 (VI. 30) Government Decree.] https://njt.hu/jogszabaly/2014-155-20-22 (2014). Accessed 17 March 2023.

[21] Maros Gy (2020). Az ImaGeo-magszkennelés módszerei egy mecseki fúrás nagy felbontású értelmezésének példáján. [Methods of ImaGeo Corescanning and a case study of a high resolution borehole evaluation from the Mecsek Mountains]. Földtani Közlöny..

[22] Dályay, V. *et al.* Dokumentációs és mintavételi terv - Fúrómag, Furadék dokumentálásának és mintázásának terve [Documentation and sampling plan – a plan for documenting and sampling drill core and drill bit.] Manuscript and attachments owned by PURAM. 118 (2014) (**in Hungarian**).

[23] Konrád Gy, Sebe K (2010). Fiatal tektonikai jelenségek új észlelései a Nyugati-Mecsekben és környezetében. [New details of young tectonic phenomena in the Western Mecsek Mts and their surroundings]. Földtani Közlöny.

[24] Lianbo Z, Jiafu Q, Yuegang L (2007). The Relationship between Fractures and Tectonic Stress Field in the Extra Low-Permeability Sandstone Reservoir at the South of Western Sichuan Depression. Journal of China University of Geosciences.

[25] Guo P, Ren D, Xue Y (2019). Simulation of multi-period tectonic stress fields and distribution prediction of tectonic fractures in tight gas reservoirs: A case study of the Tianhuan Depression in western Ordos Basin, China. Mar. Pet. Geol..

[26] Lovász, V., Karsa, R., Halász, A. & Halmai, Á. Deep Learning megoldások alkalmazhatóságának vizsgálata földtani környezetben, a Bodai Agyagkő Formáció tektonikai töréseinek példáján [Investigation of the applicability of Deep Learning solutions in a geological environment on the example of tectonic fractures of the Boda Claystone Formation] [Extended Abstract]. In *Az Elmélet és Gyakorlat Találkozása a Térinformatikában XII – Theory Meets Practice in GIS*. [Conference Book of Extended Abstracts]; 2021 Nov 11–12; Debrecen, Hungary, pp. 175–180. (Debrecen University Publisher, 2021). ISBN 978-963-318-977-1 (**in Hungarian**).

[27] Hámos G (1997). Földtani és bányászati kutatás a Nyugat-Mecseki antiklinális területén, a Bodai Formációnak, mint radioaktív hulladékbefogadó kőzetösszletnek az alkalmassága vizsgálatára. Földtani Kutatás.

[28] Konrád, Gy. Jelentés a Bodai Aleurolit Formáció 1995-1998. évi kutatásáról. Fúrás dokumentációk. Magyarázó a földtani térképhez. [Report on the Boda Aleurolite Formation research in 1995–1998. Borehole documentation. Explanatory to the geological map.] *MÉRCE Bt.* (1998) (**manuscript in Hungarian**).

[29] Maros, Gy. A Mórágyi Gránit szerkezeti fejlődése az ImaGeo magszkennerrel történt fúrásértékelések alapján. Ph.D. dissertation, University of Miskolc, Hungary. 143. http://midra.uni-miskolc.hu/document/5631/1476.pdf (2006) (**in Hungarian**).

[30] Peacock DCP, Nixon CW, Rotevatn A, Sanderson DJ, Zuluaga LF (2016). Glossary of fault and other fracture networks. J. Struct. Geol..

[31] ESRI. How Intersect works. https://pro.arcgis.com/en/pro-app/latest/tool-reference/analysis/how-intersect-analysis-works.htm. (2022). Accessed 07 November 2022.

[32] ESRI. Creating and editing multipart polygons. https://desktop.arcgis.com/en/arcmap/10.3/manage-data/editing-fundamentals/creating-and-editing-multipart-polygons.htm (2022). Accessed 07 November 2022.

[33] Maros G, Pásztor S (2001). New and oriented core evaluation method: ImaGeo. Eur. Geol..

[34] Li Y, Schmidt DR (1998). Drilling-induced core fractures and in-situ stress. J. Geophys. Res. Atmos..

[35] Konrád, Gy. Kiegészítés a BAF maganyag földtani és tektonikai dokumentálási módszertanához. *Mérce Bt.* (manuscript handed to PURAM), 32 p. (2014) (**in Hungarian**).

[36] ESRI. Introduction to deep learning. https://pro.arcgis.com/en/pro-app/latest/help/analysis/deep-learning/what-is-deep-learning-.htm (2021). Accessed 22 February 2022.

[37] Imamverdiyev Y, Sukhostat L (2019). Lithological facies classification using deep convolutional neural network. J. Pet. Sci. Eng..

[38] Zeng K, Wang Y (2020). A Deep Convolutional Neural Network for Oil Spill Detection from Spaceborne SAR Images. Remote Sensing.

[39] Guo Y, Peng S, Du W, Li D (2020). Fault and horizon automatic interpretation by CNN: a case study of coalfield. J. Geophys. Eng..

[40] He, K., Gkioxari, G., Dollár, P. & Girshick, R. Mask R‑CNN. 12 p. Preprint at arXiv: https://arxiv.org/pdf/1703.06870. 10.1109/TPAMI.2018.2844175 29994331

[41] Ghoneim, S. Accuracy, Recall, Precision, F-Score & Specificity, which to optimize on? *Towards data science.* https://towardsdatascience.com/accuracy-recall-precision-f-score-specificity-which-to-optimize-on-867d3f11124 (2021). Accessed 22 February 2022.

[42] Girshick, R. Donahue, J. Darrell, T. & Malik, J. Rich feature hierarchies for accurate object detection and semantic segmentation. p. 21. arXiv:1311.2524v5. 10.48550/arXiv.1311.2524 (2014).

[43] Paluszek, M., Thomas, S. Practical MATLAB Deep Learning—A Project Based Approach. 252 10.1007/978-1-4842-5124-9 (Apress, 2020).

[44] MathWorks. What is a Convolutional Neural Network? 3 things you need to know. Available: https://www.mathworks.com/discovery/convolutional-neural-network-matlab.html. Accessed 29 October 2022.

[45] Kim P (2017). MATLAB Deep Learning.

[46] Advanced Micro Devices, Inc. Deep Learning Training vs. Inference: What’s the Difference? https://www.xilinx.com/applications/ai-inference/difference-between-deep-learning-training-and-inference.html (2022). Accessed 08 November 2022.

[47] Linh TD, Arai M (2019). Two-stage deep neural network for general object detection. J. Inf. Process..

[48] Ren S, He K, Girshick R, Sun J (2017). Faster R-CNN: Towards real-time object detection with region proposal networks. IEEE Trans. Pattern Anal. Mach. Intell..

[49] Fu, C.Y., Shevts, M., & Berg, A.C. RetinaMask: Learning to predict masks improves state-of-the-art single-shot detection for free. 11 p. Preprint at https://arxiv.org/pdf/1901.03353.pdf, 10.48550/arXiv.1901.03353 (2019).

[50] Tran ST, Tran VP, Lee HJ, Flores JM, Le VP (2020). A two-step sequential automated crack detection and severity classification process for asphalt pavements. Int. J. Pavement Eng..

[51] He, K., Zhang, X., Ren, S. & Sun, J. Deep Residual Learning for Image Recognition [Microsoft Research], 12 p. Preprint at arXiv:1512.03385, 10.48550/arXiv.1512.03385 (2015).

[52] Yamashita R, Nishio M, Gian Do RK, Togashi K (2018). Convolutional neural networks: an overview and application in radiology. Insights Imaging.

[53] Taylor & Nitschke. Improving Deep Learning using Generic Data Augmentation [preprint] arXiv: 1708.06020 (2017), DOI: 10.48550/arXiv.1708.06020

[54] ESRI. arcgis.learn module [API]. Retrieved from https://developers.arcgis.com/python/api-reference/arcgis.learn.toc.html (2021). Accessed 22 February 2022.

[55] Han X (2021). Pre-trained models: Past, present, and future. AI Open.

[56] Baltruschat IM, Nickisch H, Grass M, Knopp T, Saalbach A (2019). Comparison of deep learning approaches for multi-label chest X-ray classification. Sci. Rep..

[57] Wu Z, Shen Ch, van den Hengel A (2019). Wider or deeper: Revisiting the ResNet model for visual recognition. Pattern Recogn..

[58] ESRI. Esri Deep Learning frameworks. Available: https://github.com/Esri/deep-learning-frameworks (2021b). Accessed 22 February 2022.

[59] ESRI. Train Deep Learning model. (Image Analyst). [Version 2.8. – archived content] https://pro.arcgis.com/en/pro-app/2.8/tool-reference/image-analyst/train-deep-learning-model.htm (2021). Accessed 22 February 2022.

[60] ESRI. Deep learning frequently asked questions. https://pro.arcgis.com/en/pro-app/2.8/help/analysis/deep-learning/deep-learning-faq.htm (2022). Accessed 13 November 2022.

[61] Shen, K. Effect of batch size on training dynamics*.* https://medium.com/mini-distill/effect-of-batch-size-on-training-dynamics-21c14f7a716e (2018). Accessed 22 February 2022.

[62] Maithani, M. Guide to Tensorflow Keras Optimizers. Developers Corner. Retrieved from https://analyticsindiamag.com/guide-to-tensorflow-keras-optimizers/ (2021). Accessed 22 February 2022.

[63] Ojha V, Timmis J, Nicosia G (2022). Assessing ranking and effectiveness of evolutionary algorithm hyperparameters using global sensitivity analysis methodologies. Swarm Evol. Comput..

[64] Kingma, D., Ba, J. L. Adam: A method for stochastic optimization. 15 p. https://arxiv.org/pdf/1412.6980.pdf, 10.48550/arXiv.1412.6980 (2015).

[65] FastAI. Optimizers. Retrieved from https://docs.fast.ai/optimizer.html (2022). Accessed 22 February 2022.

[66] PyTorch. PyTorch 1.10 documentation—Adam. https://pytorch.org/docs/stable/generated/torch.optim.Adam.html (2021). Accessed 22 February 2022.

[67] Bushaev, V. Adam—the latest trends in Deep Learning optimization. *Towards Data Science.* https://towardsdatascience.com/adam-latest-trends-in-deep-learning-optimization-6be9a291375c (2018). Accessed 22 February 2022.

[68] Brownlee, J. What is the Difference Between Test and Validation Datasets? *Machine Learning Mastery.* Retrieved from https://machinelearningmastery.com/difference-test-validation-datasets/ (2020). Accessed 22 February 2022.

[69] Russell S, Norvig P (2009). Artificial Intelligence: A Modern Approach.

[70] Kuhn M, Johnson K (2013). Applied Predictive Modeling.

[71] Sandeep, A. Object Detection – IoU – Intersection over Union. Retrieved from: https://medium.com/@nagsan16/object-detection-iou-intersection-over-union-73070cb11f6e (2019). Accessed 04 March 2022.

[72] ESRI. ArcPy. Available: https://developers.arcgis.com/documentation/arcgis-add-ins-and-automation/arcpy/ (2023). Accessed 14 February 2023.

[73] Swiderski B, Osowski S, Gwardys G (2022). Random CNN structure: tool to increase generalization ability in deep learning. J. Image Video Proc..

[74] Lawrance, S. C., Giles, C. L., & Tsoi A. C. What size neural network gives optimal generalization? Convergence properties of backpropagation. [Technical Report] *University of Maryland*, p. 35 (1996). https://clgiles.ist.psu.edu/papers/UMD-CS-TR-3617.what.size.neural.net.to.use.pdf

